# *Echinophora tenuifolia* subsp. *sibthorpiana*—Study of the Histochemical Localization of Essential Oil

**DOI:** 10.3390/molecules28072918

**Published:** 2023-03-24

**Authors:** Stanislava Ivanova, Stanislav Dyankov, Diana Karcheva-Bahchevanska, Velislava Todorova, Yoana Georgieva, Niko Benbassat, Kalin Ivanov

**Affiliations:** Department of Pharmacognosy and Pharmaceutical Chemistry, Faculty of Pharmacy, Medical University-Plovdiv, 4002 Plovdiv, Bulgaria

**Keywords:** *Echinophora*, Apiaceae, *Echinophora tenuifolia* subsp. *sibthorpiana*, *Echinophora platyloba*, *Echinophora spinosa*, folk medicine, essential oil, GC-MS

## Abstract

Background: *Echinophora tenuifolia* L. subsp. *sibthorpiana* is a perennial, aromatic plant used in traditional folk medicine and cuisine of the Mediterranean and the Middle East. However, scholars have not fully studied the pharmacological potential of the herb, and the scientific data on this plant species are limited. This study aimed to evaluate the chemical composition of the essential oil (EO) obtained from the aerial parts of *E. tenuifolia* subsp. *sibthorpiana* growing wild in Bulgaria and to perform histochemical analysis. Methods: A microscopic histochemical analysis and gas chromatography with mass spectrometry were performed. Results: The histochemical analysis confirmed the presence of terpenes in the stem and leaf of *E. tenuifolia* subsp. *sibthorpiana*. The phenylpropanoid methyleugenol was identified as the main compound in the EO, representing 48.13% of the total oil composition. There were also found considerable amounts of monoterpene hydrocarbons, representing 41.68% of the total EO. Alpha-phellandrene, *o*-cymene, and β-phellandrene were the most abundant monoterpene hydrocarbons. Conclusion: This is the first histochemical analysis performed on *E. tenuifolia* subsp. *sibthorpiana*. This is the first report of the EO composition from Bulgarian *E. tenuifolia* subsp. *sibthorpiana*, and our results indicate some future possibilities for evaluating of the biological activity of the EO of *E. tenuifolia* subsp. *sibthorpiana* and highlight the potential future use of the EO of this plant species. *E. tenuifolia* L. subsp. *sibthorpiana* EO possesses a good potential for use as a biopesticide and repellent an environmentally friendly alternative of synthetic pesticides.

## 1. Introduction

The genus *Echinophora* is a small genus within the Apiaceae family composed of about ten species distributed in the Mediterranean and the Middle East, with only three taxa (*Echinophora spinosa* L., *Echinophora tenuifolia* L. subsp. *sibthorpiana* (Guss.) Tutin, and *Echinophora tenuifolia* L. subsp. *tenuifolia*) occurring in Europe [1]. *Echinophora tenuifolia* L. subsp. *sibthorpiana* (Guss.) (*E. tenuifolia* subsp. *sibthorpiana*) is the only representative of the genus in the Bulgarian flora [2,3]. It is a perennial, aromatic plant with a hard stem reaching up to 50 cm in height, branching almost from the base and densely covered with trichomes. The basal leaves have petioles with lobes two to three times deeper than the shoulders. The apical leaves are sessile, and the flowers are yellow, arranged in an inflorescence compound umbel on the terminal branches of the stems [3]. The species grows in other countries, most notably in Turkey, where people use it as a spice in pickles, meatballs, and the traditional fermented cereal food Tarhana [4]. *E. tenuifolia* subsp. *sibthorpiana* is also known as Tarhana herb. According to some sources, Tarhana is one of the oldest foods in the cuisine of the Eastern Mediterranean [5,6]. In Greek cuisine, it is known as Trahanas [5]. This fermented cereal-based food is prepared by mixing yoghurt, cereal flours, yeast, different vegetables, herbs, and spices. After mixing the main ingredients, the dough ferments for several days (usually up to 5) [6]. The last step of the process is drying. The specific sour taste and yeast flavor of Tarhana is due to the presence of lactic acid bacteria and yeast [6]. Although *E. tenuifolia* subsp. *sibthorpiana* is used as a spice in the production of the Tarhana, it also plays an essential role in the fermentation process [4]. People also use *E. tenuifolia* L. subsp. *sibthorpiana* as a folk medicine for its wound-healing properties, its digestive activity, and for gastric ulcer treatment [7]. 

Plants used in nutrition and folk medicine are particularly rich in many bioactive compounds, possessing various biological effects, and some of them have a promising potential for inclusion in innovative herbal medicines or functional foods. The beneficial effects of these plants are due to the whole, rather than any single nutrient or functional compound [8,9].

However, scholars have not fully studied the pharmacological potential of the herb, and the scientific data on this plant are limited. The authors of several studies have investigated the pharmacological properties of species from the genus *Echinophora*, and Table 1 summarizes these studies.

*E. tenuifolia* L. subsp. *sibthorpiana* could be considered not only as a plant used in nutrition and traditional medicine but also as a source of essential oil.

Essential oils are important sources of compounds with health-beneficial activity in humans, while their main function in plants is to protect them from insects or pathogenic microorganisms [23]. The concentration of the different volatile compounds in essential oils is strongly influenced not only by the plant species but also by the geographical region, the harvesting process, and the soil type [23].

Several previous phytochemical studies have investigated the composition of the *E. tenuifolia* L. subsp. *sibthorpiana* EO. The major compounds identified in the EO are the phenylpropanoid methyleugenol (ME) and the monoterpene hydrocarbons α-phellandrene, δ-3-carene, and *p*-cymene [1,22,24,25,26,27,28]. Different factors including the geographical location, collection time, harvest year, growth stage of the plant, and postharvest handling, specifically the drying method and the EO storage, considerably affect the chemical composition of the EO [7,24,29,30]. When reviewing the literature, no records that discussed the composition of the *E. tenuifolia* subsp. *sibthorpiana* EO from Bulgaria were found. Additionally, no record of this aromatic plant exists in any standardization document.

The aim of the present study was to evaluate the chemical composition of the EO from aerial parts of *E. tenuifolia* subsp. *sibthorpiana* growing wild in Bulgaria and to perform histochemical analysis. The histochemical analysis of *E. tenuifolia* subsp. *sibthorpiana* has been carried out for the first time. Its aim was to trace the accumulation of terpenes, in particular the essential oil (composed mainly of monoterpenes and sesquiterpenes), as a secondary metabolite.

## 2. Results and Discussion

### 2.1. Microscopic Histochemical Analysis

**Figure 1:**
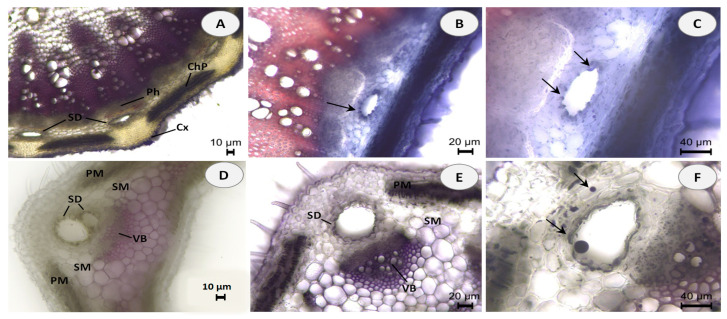
Histochemical observations of terpenes stained with NADI reagent in stem and leaf cross sections of *E. tenuifolia* L. subsp. sibthorpiana (Guss.) Tutin. (**A**) General aspect of stem section, showing the presence of secretory ducts (SD) arranged in a ring between the cortex (Cx), secondary phloem (Ph) and chlorophyllous parenchyma (ChP). (**B**,**C**) Details of stem secretory ducts (SD) with positive blue staining droplets. (**D**) General aspect of leaf, showing palisade mesophyll (PM) and spongy *mesophyll* (SM) with secretory ducts (SD) adjacent to the vascular bundles (VB). (**E**,**F**) Details of leaf secretory ducts (SD) with positive blue staining of the one-layered epithelium and droplets in the intercellular. Scale bars: (**A**,**D**) = 10 µm; (**B**,**E**) = 20 µm; (**C**,**F**) = 40 µm.

**Figure 2:**
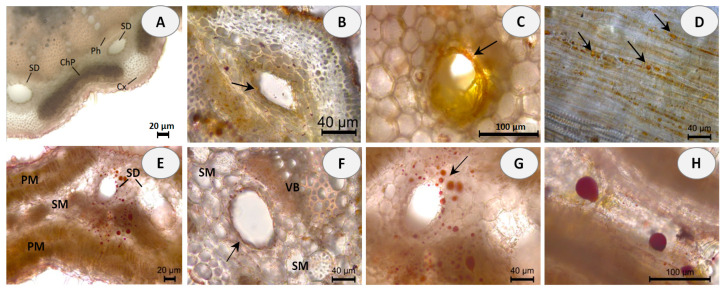
Histochemical observations of lipids stained with Sudan III in stem and leaf sections of *E. tenuifolia* L. subsp. *sibthorpiana* (Guss.) Tutin. (**A**) General aspect of transverse stem section, showing the presence of secretory ducts (SD) between the cortex (Cx), secondary phloem (Ph) and chlorophyllous parenchyma (ChP). (**B**,**C**) Details of stem secretory ducts with positive red-orange staining of the surrounding epithelial cells. (**D**) Tangential longitudinal section of the cortical region, showing the presence of lipid droplets with positive red-orange staining. (**E**) Leaf cross section, showing the presence of secretory ducts (SD) with red stained lipid droplets, located in the spongy mesophyll (SM) and palisade mesophyll (PM). (**F**) Positive red staining of the epithelial cells around the leaf ducts. (**G**,**H**) Details of leaf secretory ducts with positive red staining of the lipid deposits. Scale bars: (**A**,**E**) = 20 µm; (**B**,**D**,**F**,**G**) = 40 µm; (**C**,**H**) = 100 µm.

Hand-cut transverse and longitudinal sections of the stem and leaf of *E. tenuifolia* subsp. *sibthorpiana* were stained with NADI reagent to detect terpenes and Sudan III to detect lipids. NADI reagent produces differential staining in plant histology to demonstrate various terpene classes. The staining is blue with essential oils and red with resins (diterpenes, triterpenes, and derivatives) [26]. Positive blue staining indicates the presence of essential oil, containing mainly mono- and sesquiterpenes (Figure 1). In the Sudan III test, the presence of red or orange staining demonstrates the presence of lipid compounds (Figure 2).

Essential oils can be produced in multiple secretory structures, including those that are external, which involve secretory glandular trichomes, and those that are internal, which include secretory cavities, ducts, and individual essential oil cells (parenchyma cells that secrete and store essential oil). These secretory structures can vary depending on the botanical family. The secretory ducts in the plant family Apiaceae are a typical example of schizogenous cavities.

In the present histochemical analysis of *E. tenuifolia* subsp. *sibthorpiana*, such internal secretory ducts were observed in both of the plant organs examined. In the stem, these oil secretory structures were arranged in a ring between the phloem parenchyma and the surrounding cortex (Figure 1A and Figure 2A). In the leaf, the ducts were located among the mesophyll cells, accompanying the vascular bundles (Figure 1D,E and Figure 2E,F). The NADI reaction resulted in blue staining of the secreted droplets in the ducts and related surrounding cells in the samples of both plant organs analyzed, which confirmed the presence of essential oil (Figure 1B,C,E,F). Regarding the Sudan III test, the lipid nature of the secretory ducts was confirmed by the positive red-orange staining of the epithelial cells, grouped as a layer around the duct and responsible for the oil secretion (Figure 2B,C,F). In addition, numerous red-orange stained lipid droplets were observed in the leaf mesophyll cells adjacent to the ducts (Figure 2G,H), as well as in the tangential stem section of the cortical region (Figure 2D). Due to the lipophilic nature of essential oil compounds, their presence was confirmed in both analyses.

### 2.2. Volatile Constituents of the E. tenuifolia ssp. sibthorpiana Essential Oil

**Figure 3:**
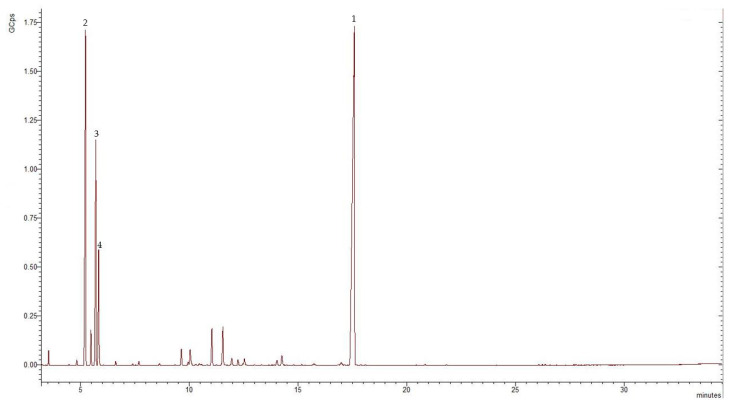
GC-MS chromatogram of the *E. tenuifolia* subsp. *sibthorpiana* EO, where GCps—Giga Counts per second, and the numbers refer to the following compounds: 1—methyleugenol, 2—α-phellandrene, 3—o-cymene, and 4—β-phellandrene.

The essential oil obtained from the aerial parts of *E. tenuifolia* subsp. *sibthorpiana* was pale yellow in color with a 1.3% yield. The results of the GC-MS analyses of the essential oil showed that 16 constituents representing 92% of the total oil content were tentatively present. Figure 3 and Table 2 show the chromatogram and the chemical composition of the EO.

The identified compounds belonged to the phenylpropanoids, the monoterpene hydrocarbon, and the oxygenated monoterpene classes. Phenylpropanoid methyleugenol was the main compound identified in the analyzed EO, representing 48.13% of the total oil composition. Considerable amounts of monoterpene hydrocarbons were also found, representing 41.68% of the total EO. Alpha-phellandrene, *o*-cymene, and β-phellandrene were the most abundant monoterpene hydrocarbons, representing 20.51%, 12.29%, and 6% of the total oil, respectively. The oxygenated monoterpenes represented 2.22% of the total EO.

Our results complied with the results of earlier studies on the chemical composition of *E. tenuifolia* subsp. *sibthorpiana* EO from different geographical regions (Table 3).

The data suggest that the main volatile compounds identified in E. sibthorpiana from some regions of Turkey were methyleugenol and α-phelandrene. The amount of methyleugenol ranges from 9.61% to 90.16%, and that of α-phellandrene ranges from 6.10% to 66.39% [22,24,25,28,29,30,32,33] which corresponds with the results of the current study. Methyleugenol has also been identified as a major compound in the EO derived from the plant species studied, distributed in some regions in Iran (50.40%) and the Republic of North Macedonia (60.40%) [26,27]. Additionally, in previous reported studies, α-phellandrene (10.23–43.80%) was among the major volatile compounds detected in *E. tenuifolia* subsp. *sibthorpiana* EO from regions of Iran, Macedonia, and Greece [1,26,27,31]. Despite the different factors such as harvest period, environmental conditions, and geographical variations affecting the chemical composition of EO and the observed quantitative differences, the main constituent detected in EOs from Bulgaria, Turkey, and Macedonia was methyleugenol [1,24,25,26,34]. The monoterpene hydrocarbon δ-3-carene has been identified as a major constituent of EO from Turkey [22]; however, this compound was not detected in the presented EO analysis. Sesquiterpenes were also not detected. While in small amounts the sesquiterpenes germacrene D, β-selinene, β-ionone, and caryophyllene oxide were identified in previous studies [25,26].

Compared to other European species of the genus *Echinophora*, the main constituents found in *E. spinosa* from Montenegro were δ-3-carene, α-phellandrene, and *p*-cymene [35]. Beta-phellandrene was found as the major constituent of flowering aerial parts of *E. spinosa* from Italy, with myristicin, *p*-cymene, and δ-3-carene also being present in significant amounts. The major volatile constituent of EO obtained from the ripe fruit of the same plant was *p*-cymen [18]. Para-cymen, α-phellandrene, and α-pinene were the principal constituents of EO of the aerial parts of *E. spinosa* from Corsica Island, France. Methyleugenol was presented in small amounts in the root EO of the same plant, while the most abundant compound was the phenylpropanoid myristicin [36].

The major volatile compounds identified in EO from Iranian *E. platyloba* were trans-β-ocimene, β-cymene spathulenol, β-myrcene, DL-limonene, α-pinene, β-phellandrene, and α- phellandrene [37,38,39]. A study on the chemical composition of EO isolated from *Echinophora lamondiana* B. Yildiz et Z. Bahcecioglu showed that the major volatile compounds were δ-3-carene, α-phellandrene, and *p*-cymene [40,41]. In a previous study, it was reported that the EO of *E. tournefortii* contained α-pinene and caryophyllene oxide as the major volatile components, while the EO of *E. chrysantha* contained α-phellandrene and β-phellandrene [42]. Moreover, it has been reported that the *E. trichophylla* EO contained sabinene and 2,6-dimethyl-1,3(E),5(E),7-octatetraene as main constituents, that the EO of *E. carvifolia* contained β-caryophyllene and germacrene D, and that the EO of *E. orientalis* contained myrcene and *p*-cymene as major volatile components [42].

When comparing EOs isolated from different *Echinophora* species, the main volatile compounds detected were α-phellandrene, β-phellandrene, δ-3-carene, myrcene, and *p*-cymene. The difference between EO isolated from *E. tenuifolia* subsp. *sibthorpiana* and EO isolated from other *Echinophora* species was the detected methyleugenol as the main volatile compound in the *E. tenuifolia* subsp. *sibthorpiana* EO.

Methyleugenol represents a colorless to pale yellow liquid with a clove odor and a bitter taste [43]. It could be produced by methylation of eugenol or isolated from the EO of different plants such as citronella (*Cymbopogon* spp.), basil (*Ocimum* spp.), and tea tree (*Melaleuca* spp.) [43]. Nowadays, it is mainly used as repellent [43]. In 1975, ME was classified by the Food and Drug Administration (FDA) as a safe compound under the conditions of use. It was approved for use in foods as a flavoring agent and as a fragrance in cosmetics. However, the safety of ME is a matter of some controversy [44]. The biological activity of ME is quite diverse and has been assessed by various in vitro and in vivo studies [45,46,47,48,49,50,51,52,53,54,55]. Methyleugenol exerted antiallergic activity in IgE-activated RBL-2H3 cells through various mechanisms [45,46]. It showed neuroprotective effects due to its radical scavenging activity and inhibition of the production of NO and proinflammatory cytokines [47]. Methyleugenol possessed counter-anorexigenic effects related to satiety [50]. The central effects of ME may be attributed to its agonism toward inhibitory neuronal γ-aminobutyric acid (GABA) receptors [48,49,51]. Another study reported the potential of ME to lower blood pressure, most likely by relaxation of the vascular smooth muscles [53]. It has been reported that methyleugenol has significant antifungal activity [54,55]. Methyleugenol also demonstrated antibacterial activity against *Staphylococcus aureus*, *Streptococcus pneumoniae*, and *Haemophilus influenzae* [54]. Methyleugenol intake was also associated with anesthetic, hypothermic, myorelaxant, and anticonvulsant effects [52]. However, it should be noted that cytotoxicity, genotoxicity, and hepatotoxicity have also been reported in animal studies [56,57,58]. The lipophilicity of phenylpropanoids had an influence on their toxicity [54]. Additionally, the toxic effects are dose-dependent. The cancerogenic doses of methyleugenol in rodents following chronic exposure are approximately 1000-fold higher than the usual dietary intake of methyleugenol as a flavoring constituent in food [59]. According to the European Medicine Agency (EMA) report, the acute toxicity of this compound has not been fully investigated [44]. EMA reports LD50 values for ME from 850 to 1560 mg/kg for rats and from 540 mg/kg for mice.

**Figure 4:**
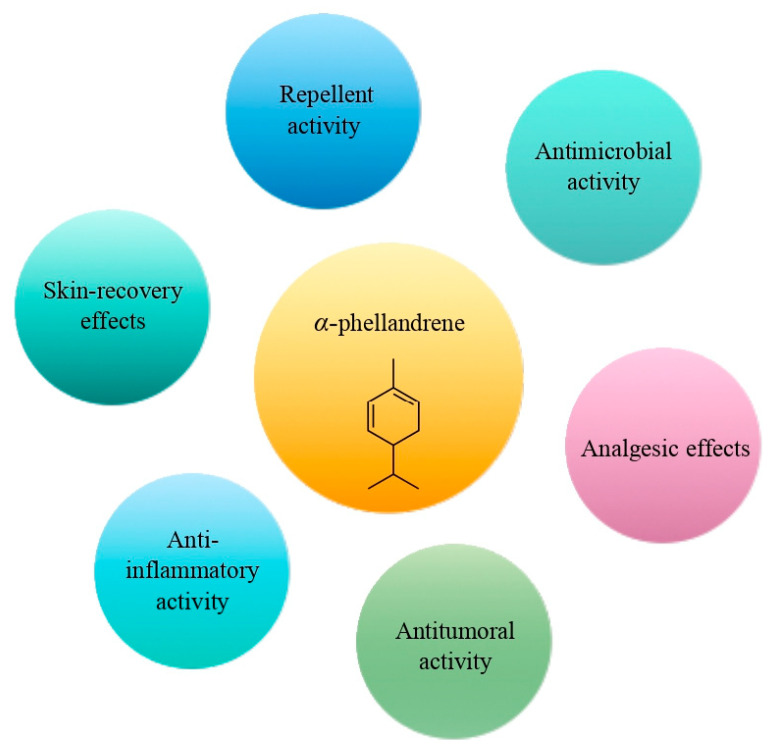
The main effects of α-phellandrene.

The cyclic monoterpene α-phellandrene was introduced for public use in the 1940s [60]. It represents a colorless liquid, characterized by a peppery, woody, and herbaceous aroma [60]. It could be found in cosmetics, food, and EOs. Nowadays, α-phellandrene has been characterized by a broad spectrum of pharmacological activities, including antimicrobial, anti-inflammatory, antitumoral, analgesic, and insecticidal activities (Figure 4) [60,61,62,63]. Alpha-phellandrene accelerated wound closure due to collagen deposition and demonstrated good wound-healing properties [64,65,66]. In addition, α-phellandrene is associated with enhancement of the proliferation of vascular endothelial growth factor in dermal papilla cells, which indicates that α-phellandrene has a strong potential to prevent hair loss [67]. According to recent studies, α-phellandrene could be used as a preservant in the food industry [68,69]. 

Scholars have mainly investigated the biological activity of *o*-cymene and β-phellandrene as components of different essential oils [67,70,71,72,73,74]. No randomized double-blind studies involving methyleugenol, α-phellandrene, *o*-cymene, and β-phellandrene exist. Such studies would be useful to confirm the safety and future applications of these compounds as medicines. In our view, the EO isolated from *E. tenuifolia* subsp. *sibthorpiana* has a good potential to be included in innovative herbal medicinal products, functional foods, and food supplements aimed at improving chronic mood disorders, cognitive impairment, skin recovery, etc. However, studies on this EO are limited; in vitro and in vivo studies would be especially beneficial for evaluation of its real potential. The acute and the sub-chronic toxicity of this EO should also be well investigated.

Nowadays, there is a great interest in EOs as products for pest management and repellents [75] because of their much safer potential than synthetic pesticides. Something more, EOs are a green alternative tool for insect management that is less environmentally damaging [75,76]. *E. tenuifolia* L. subsp. *sibthorpiana* EO could be a promising species for use as a biopesticide and a biorepellent because of the concentrations of its active compounds: methyleugenol and α-phellandrene.

## 3. Materials and Methods

### 3.1. Plant Material

The aerial parts of flowering *E. tenuifolia* L. subsp. *sibthorpiana* were collected in August 2022 near the village of Raykova Mogila (41°50′14.9″ N 26°18′17.3″ E), Thracian Lowland floristic region, Bulgaria. A voucher specimen of the species (No: 063306) was deposited in the Herbarium of the Agricultural University—Plovdiv (SOA).

### 3.2. Chemicals and Reagents

To determine the retention indices (RI), the following hydrocarbons were used: nonane (≥99%), decane (≥99%), undecane (≥99%), dodecane (99%), tridecane (≥99%), tetradecane (≥99%), and hexadecane (≥99%) purchased from Merck KGaA (Darmstadt, Germany). Dichloromethane (Sigma Aldrich, Steinheim, Germany) was used for the dilution of the EO.

### 3.3. Microscopic Histochemical Analysis

The plant material had been fixed in a solution of 50% ethanol and glycerol in a ratio of 7:3 for 24 h to soften the tissues. Hand-cut transverse and tangential longitudinal sections were made from the stem and leaf of the plant. Terpene histochemistry was examined using NADI reagent (0.5 mL of 0.1% α-naphthol (Merck KGaA, Darmstadt, Germany), 0.5 mL of 1% *N*,*N*-dimethyl-*p*-phenylenediamine (Merck KGaA, Darmstadt, Germany), and 49 mL of 0.1 M sodium phosphate buffer, pH 7.2 (Merck KGaA, Darmstadt, Germany) [77]. Cross sections were incubated with NADI reagent for 1 h in the dark, washed in sodium phosphate buffer (0.1 M, pH 7.2) for 2 min, and mounted in the same buffer. Lipid histochemistry was examined using Sudan III (Merck KGaA, Darmstadt, Germany). The sections were treated with Sudan III in 70% ethanol for 30 min, rinsed briefly in 80% ethanol, and mounted in 50% glycerol [77]. The sections were observed with a light microscope (Leica DM 2000 LED, Leica Microsystems, Wetzlar, Germany) equipped with a digital camera (Leica DMC 2900) and image processing software (Leica Application Suite, LAS).

### 3.4. Isolation of the Essential Oil

The air-dried flowering aerial parts (100 g) of the plant were subjected to hydrodistillation for 4 h using a Clevenger-type apparatus to obtain the essential oil. The collected oil was dried over anhydrous sodium sulfate and stored in dark glass vials at 4 °C until the GC-MS analysis. The oil yield (%) was measured based on the dry weight of the plant.

### 3.5. Chromatographic Conditions

The analysis of the EO was performed using gas chromatography with mass spectrometry (GC-MS). For the GC-MS analysis a Bruker Scion 436-GC SQ MS (Bremen, Germany) equipped with a Bruker BR-5ms fused silica capillary column (0.25 µm film thickness and 15 m × 0.25 mm i.d.) was used. Helium was used as a carrier gas with a constant flow rate of 1mL/min. The injection volume was 1 µL. The split ratio of the injector was 1:50. The oven temperature was set at 50 °C for 1 min, then increased to 140 °C at a rate of 4 °C/min, and then increased to 290 °C at a rate of 15 °C/min and held for 1 min. The temperature of the injector was set to 250 °C, and the detector temperature was set to 300 °C. The mass spectra were in full scan mode with a mass range of 50–350 *m*/*z*. The identification of the separated essential oil constituents was achieved by comparing their MS spectra and retention indices (RI) with spectral data within the Wiley NIST11 Mass Spectral Library (NIST11/2011/EPA/NIH) and the literature data. The RI values were calculated from the retention times of the C_8_–C_30_ *n*-alkane series injected under the same conditions described above.

## 4. Conclusions

This is the first report describing the histochemical localization of *Echinophora tenuifolia* subsp. *sibthorpiana* EO and the first study which investigated the chemical profile of this EO from Bulgarian *E. tenuifolia* L. subsp. *sibthorpiana*. The presented histochemical analysis confirmed the presence of lipids and terpenes in the stem and leaf of *E. tenuifolia* subsp. *sibthorpiana*. The conducted GC-MS analysis indicated the presence of monoterpene hydrocarbons, oxygenated monoterpenes, and phenylpropanoid. Sixteen volatile constituents representing 92.03% of the total oil were tentatively detected. The major component of the isolated EO was methyleugenol, representing 48.13%. The results obtained in this study indicated that future possibilities to evaluate the biological activity of the EO of *E. tenuifolia* L. subsp. *sibthorpiana* exist, and they highlight the potential future use of the EO of this plant species. Further investigations in this area could be especially useful for the future development of novel medicines for use in cardiology, neurology, rheumatology, and psychiatry. *E. tenuifolia* L. subsp. *sibthorpiana* EO also has a prominent potential for use as a biopesticide and repellent as well.

## Figures and Tables

**Table 1:** Studies investigating the biological and pharmacological activities involving species from the genus *Echinophora*.

Study Objectives	Study Design	Main Results	Ref.
*Echinophora platyloba* (*E. platyloba*) (ethanolic extract)	In vitro testing for anti-*Candida albicans* activity	*E. platyloba* extract, in a concentration ≥ 2 mg/mL, effectively inhibited *Candida albicans* growth.	[10]
*Echinophora platyloba*	Double-blind randomized clinical trial. The study included 60 women diagnosed with candida albicans vaginitis. The authors randomized patients into two groups: an *Echinophora* cream plus fluconazole group and a fluconazole group.	The cream containing *E. platyloba* extract was effective at reducing candida vaginitis in women with the disease.	[11]
*Echinophora platyloba* (extract)	Single-blind randomized clinical trial. The study included 90 women diagnosed with moderate-to-severe premenstrual syndrome (PMS). The authors randomized participants into three groups: an *Echinophora-platyloba* extract group, a fennel extract group, and a placebo group.	The *E. platyloba* and fennel extracts reduced the severity of PMS.	[12]
*Echinophora platyloba* (ethanolic extract)	In vivo evaluation of hepatoprotective activity against acetaminophen-induced hepatotoxicity in rats.	*E. platyloba* extract pretreatment showed potent hepatoprotective effects.	[13]
*Echinophora platyloba* (methanolic extract)	In vitro evaluation of cytotoxicity and the mechanism of cell death against the human prostate adenocarcinoma PC 3 and human umbilical vein endothelial cells (HUVEC) cell line.	*E. platyloba* extract inhibited the human prostate adenocarcinoma cell proliferation possibly via apoptosis and might be valuable for human prostate adenocarcinoma treatment.	[14]
*Echinophora platyloba* (methanolic extract)	In vitro examination of the cytotoxic activity and mechanism of the cell death of *E.platyloba* methanolic extracts on mouse fibrosarcoma cells (WEHI-164).	The extract time- and dose-dependently inhibited the proliferation of fibrosarcoma cells possibly via an apoptosis-dependent pathway.	[15]
*Echinophora cinerea* (*E. cinerea*) (extract)	In vitro investigation of antiprotozoal activity of *E. cinerea* against *Giardia lamblia* and *Giardia muris*, as well as the cytotoxicity of this plant extract.	*E. cinerea* showed considerable antigiardial activity against *G. lamblia* and exerted low cytotoxicity on the studied cell lines.	[16]
*Echinophora cinerea*	In vitro assessment of the protective effect of six compounds isolated from *E. cinerea* (quercetrin-3-*O*-β-d-glucopyranoside, osthol, verbenone-5-*O*-β-d-glycopyranoside, Isoimperatorin, kaempferol-3-*O*-β-d-glucopyranoside, and echinophorin B) against oxidative stress and apoptosis induced by cisplatin in PC12 cells.	Quercetrin-3-*O*-β-d-glucopyranoside and osthol showed apoptosis inhibition and effectively blocked the cisplatin-induced neurotoxicity.	[17]
*Echinophora spinosa* L. (*E. spinosa*) aerial parts and ripe fruits (essential oil)	In vitro determination of the antimicrobial activity of the two essential oils against some bacteria responsible for intestinal dysbiosis.	The *E. spinosa* essential oils showed selective antibacterial activity against potentially pathogenic intestinal bacteria such as *Clostridium difficile*, *Clostridium perfringens, Enterococcus* *faecalis*, *Eubacterium limosum*, *Peptostreptococcus anaerobius*, and the fungus *Candida albicans*, and they were less active against bifidobacteria and lactobacilli.	[18]
*Echinophora tournefortii* Jaub. and Spach (*E. tournefortii*) (methanol, acetone, and water extracts)	In vitro examination of the antioxidant capacities of the extracts using six complementary methods and cytotoxic activity.	Generally, all the extracts showed strong antioxidant activity and exhibited cytotoxic activities.	[19]
*Echinophora tenuifolia* L. (*E. tenifolia*) inflorescence (methanolic extract)	In vitro investigation of antioxidant and anti-inflammatory activities.	The *n*-hexane fraction and the dichloromethane fraction of the extract showed considerable anti-inflammatory activity	[20]
*Echinophora tenuifolia* L. branches (methanolic extract)	In vitro investigation of the cell growth inhibitory activity on different human cancer cell lines and normal BJ fibroblasts	All the samples showed effectivity against the melanoma cell line C32, with IC50 values from 22.8 ± 0.8 to 78.7 ± 1.2 µg/mL, with the dichloromethane fraction activity being the highest.	[21]
*Echinophora tenuifolia* L. subsp. *sibthorpiana* (essential oil)	In vitro investigation of the antioxidant and antimicrobial activities	The essential oil showed antioxidant as well as antimicrobial effects, especially against *Bacillus cereus* and *Staphylocoocus* spp.	[22]

**Table 2:** Volatile constituents of the EO of *E. tenuifolia* subsp. *sibthorpiana* from Bulgaria as a percentage of total EO; where tr—trace (less than 0.05%).

No.	Compound	RI	Formula	Class of Compound	% of Total EO
1	α-thujene	947	C_10_H_16_	MH	tr
2	α-pinene	952	C_10_H_16_	MH	0.56
3	β-pinene	982	C_10_H_16_	MH	tr
4	β-myrcene	993	C_10_H_16_	MH	0.24
5	α-phellandrene	1007	C_10_H_16_	MH	20.51
6	α-terpinene	1014	C_10_H_16_	MH	1.66
7	*o*-cymene	1022	C_10_H_14_	MH	12.30
8	β-phellandrene	1026	C_10_H_16_	MH	6.0
9	γ-terpinene	1051	C_10_H_16_	MH	0.20
10	α-terpinolene	1085	C_10_H_16_	MH	0.21
11	β-terpineol	1149	C_10_H_18_O	MO	0.96
12	cryptone	1175	C_9_H_14_O	MO	0.12
13	*p*-ment-1(7)-en-2-one	1240	C_10_H_16_O	MO	0.08
14	isocarveol	1290	C_10_H_16_O	MO	0.35
15	carvacrol	1298	C_10_H_14_O	MO	0.71
16	methyleugenol	1405	C_11_H_14_O	PP	48.13
	Terpene classes				
	MH—monoterpene hydrocarbons				41.68
	MO—oxygenated monoterpenes				2.22
	PP—phenylpropanoids				48.13
	Total identified				92.03

Relative peak area percentage is shown as mean of three independent measurements and the standard error of the mean has been removed and does not exceed 2%.

**Table 3:** Comparison of the main volatile constituents of the EO of *E. tenuifolia* subsp. *sibthorpiana* from different regions.

Plants Collecting Region	Main Volatile Compounds %	Other Volatile Compounds	Ref.
Bulgaria	methyleugenol (48.13%)	α-phellandrene (20.51%) *o*-cymene (12.30%)	Present study
Greece	α-phellandrene (43.80%)	methyleugenol (28.60%)	[1]
Iran	methyleugenol (50.40%)	α-phellandrene (16.30%) δ-3-carene (17.40%)	[27]
Iran	δ-3-carene (31.80%) α-phellandrene (31.00%)	methyleugenol (16.90%)	[31]
Republic of North Macedonia	methyleugenol (60.40%)	*p*-cymene (11.18%) α-phellandrene (10.23%)	[26]
Turkey	α-phellandrene (47.43–66.39%) methyl eugenol (21.29–38.72%)		[7]
Turkey	δ-3-carene (17.93%) methyleugenol (16.41%)	*p*-cymene (8.99%) α-phellandrene (9.33%)	[22]
Turkey	δ-3-carene (30.01–38.80%)	methyleugenol (22.10–25.96%) α-phellandrene (14.50–29.26%)	[24]
Turkey	methyleugenol (41.80–62.90%)	α-phellandrene (30.40%) *p*-cymene (7.80–9.10%)	[25]
Turkey	methyleugenol (36.60%) δ-3-carene (36.60%)	*p*-cymene (7.60%) α-phellandrene (6.10%)	[28]
Turkey	methyleugenol (9.61–80.65%) δ-3-carene (2.27–61.64%)		[29]
Turkey	methyleugenol (24.99–90.16%) δ-3-carene (2.57–34.80%)	*p*-cymene (1.23–9.81%)	[30]
Turkey	α-phellandrene (51.52%)	methyleugenol (17.46%) *p*-cymene (14.66%)	[32]
Turkey	α-phellandrene (13.22–55.27%) δ-3-carene (49.29–4.03%)	methyleugenol (22.59–25.69%)	[33]

## Data Availability

Not applicable.

## Sample Availability

Samples of the essential oil are not available.
